# Supplementary material for: Modeling glioblastoma heterogeneity as a dynamic network of cell states
Source: Mol Syst Biol. 2021 Sep 16;17(9):e10105. doi: 10.15252/msb.202010105 (PMC8444284; doi:10.15252/msb.202010105)
Supplement: Supplementary file 5 — Source Data for Figure 3 [file MSB-17-e10105-s001.zip › Figure3A_sourcedata/GSEA_3065/nosigdb_state5.GseaPreranked.1622718435914/RICHARDS_ZHONG_NATURE_2018_ASTROCYTES_UPREG.html]

Details for gene set RICHARDS\_ZHONG\_NATURE\_2018\_ASTROCYTES\_UPREG[GSEA]

|  || Dataset | state5 |
| Phenotype | NoPhenotypeAvailable |
| Upregulated in class | na\_pos |
| GeneSet | RICHARDS\_ZHONG\_NATURE\_2018\_ASTROCYTES\_UPREG |
| Enrichment Score (ES) | 0.38510728 |
| Normalized Enrichment Score (NES) | 1.7668598 |
| Nominal p-value | 0.0 |
| FDR q-value | 0.012290858 |
| FWER p-Value | 0.105 |
Table: GSEA Results Summary

  

Fig 1: Enrichment plot: RICHARDS\_ZHONG\_NATURE\_2018\_ASTROCYTES\_UPREG      
 Profile of the Running ES Score & Positions of GeneSet Members on the Rank Ordered List

  

| PROBE | GENE SYMBOL | GENE\_TITLE | RANK IN GENE LIST | RANK METRIC SCORE | RUNNING ES | CORE ENRICHMENT || 1 | PTPRZ1 |  |  | 12 | 0.862 | 0.0137 | Yes |
| 2 | CD9 |  |  | 14 | 0.857 | 0.0286 | Yes |
| 3 | BCAN |  |  | 15 | 0.850 | 0.0435 | Yes |
| 4 | PTN |  |  | 19 | 0.824 | 0.0576 | Yes |
| 5 | CD63 |  |  | 24 | 0.769 | 0.0706 | Yes |
| 6 | SCRG1 |  |  | 36 | 0.715 | 0.0819 | Yes |
| 7 | GPM6B |  |  | 41 | 0.702 | 0.0937 | Yes |
| 8 | TNC |  |  | 45 | 0.680 | 0.1053 | Yes |
| 9 | B2M |  |  | 61 | 0.601 | 0.1141 | Yes |
| 10 | ADGRG1 |  |  | 74 | 0.567 | 0.1227 | Yes |
| 11 | DDR1 |  |  | 81 | 0.551 | 0.1316 | Yes |
| 12 | SLC1A3 |  |  | 82 | 0.550 | 0.1412 | Yes |
| 13 | PON2 |  |  | 84 | 0.546 | 0.1507 | Yes |
| 14 | MSMO1 |  |  | 92 | 0.540 | 0.1594 | Yes |
| 15 | PRCP |  |  | 103 | 0.523 | 0.1674 | Yes |
| 16 | TIMP1 |  |  | 109 | 0.518 | 0.1759 | Yes |
| 17 | ITGAV |  |  | 118 | 0.508 | 0.1839 | Yes |
| 18 | BCAP29 |  |  | 127 | 0.502 | 0.1917 | Yes |
| 19 | TTYH1 |  |  | 141 | 0.489 | 0.1988 | Yes |
| 20 | FAT1 |  |  | 155 | 0.478 | 0.2057 | Yes |
| 21 | CLU |  |  | 162 | 0.474 | 0.2133 | Yes |
| 22 | PTTG1IP |  |  | 214 | 0.430 | 0.2150 | Yes |
| 23 | RCN1 |  |  | 217 | 0.427 | 0.2223 | Yes |
| 24 | SLC3A2 |  |  | 222 | 0.424 | 0.2293 | Yes |
| 25 | ALCAM |  |  | 225 | 0.422 | 0.2364 | Yes |
| 26 | SYPL1 |  |  | 231 | 0.418 | 0.2432 | Yes |
| 27 | MFGE8 |  |  | 233 | 0.418 | 0.2504 | Yes |
| 28 | SERPINE2 |  |  | 238 | 0.414 | 0.2572 | Yes |
| 29 | SCD |  |  | 284 | 0.391 | 0.2589 | Yes |
| 30 | HSD17B12 |  |  | 311 | 0.377 | 0.2625 | Yes |
| 31 | TMEM132B |  |  | 335 | 0.365 | 0.2663 | Yes |
| 32 | SFXN5 |  |  | 342 | 0.364 | 0.2720 | Yes |
| 33 | LGALS3BP |  |  | 349 | 0.361 | 0.2776 | Yes |
| 34 | TM7SF2 |  |  | 352 | 0.360 | 0.2837 | Yes |
| 35 | DAG1 |  |  | 373 | 0.351 | 0.2876 | Yes |
| 36 | DHCR7 |  |  | 387 | 0.347 | 0.2922 | Yes |
| 37 | SEZ6L |  |  | 422 | 0.334 | 0.2941 | Yes |
| 38 | BCHE |  |  | 425 | 0.333 | 0.2997 | Yes |
| 39 | ABHD3 |  |  | 458 | 0.323 | 0.3017 | Yes |
| 40 | AASS |  |  | 494 | 0.313 | 0.3032 | Yes |
| 41 | RFX4 |  |  | 509 | 0.308 | 0.3070 | Yes |
| 42 | EDNRB |  |  | 519 | 0.306 | 0.3113 | Yes |
| 43 | MRC2 |  |  | 543 | 0.299 | 0.3140 | Yes |
| 44 | ITM2C |  |  | 551 | 0.297 | 0.3184 | Yes |
| 45 | LAMB2 |  |  | 578 | 0.290 | 0.3205 | Yes |
| 46 | NTRK2 |  |  | 587 | 0.288 | 0.3246 | Yes |
| 47 | MLC1 |  |  | 591 | 0.288 | 0.3293 | Yes |
| 48 | SLC4A4 |  |  | 598 | 0.286 | 0.3336 | Yes |
| 49 | TEX264 |  |  | 620 | 0.281 | 0.3362 | Yes |
| 50 | PMP22 |  |  | 663 | 0.272 | 0.3361 | Yes |
| 51 | DNER |  |  | 671 | 0.270 | 0.3401 | Yes |
| 52 | HTRA1 |  |  | 690 | 0.266 | 0.3427 | Yes |
| 53 | PDPN |  |  | 719 | 0.258 | 0.3440 | Yes |
| 54 | G6PC3 |  |  | 726 | 0.256 | 0.3478 | Yes |
| 55 | SDC3 |  |  | 728 | 0.255 | 0.3521 | Yes |
| 56 | LRIG1 |  |  | 754 | 0.250 | 0.3537 | Yes |
| 57 | NOTCH2 |  |  | 756 | 0.249 | 0.3579 | Yes |
| 58 | FADS2 |  |  | 780 | 0.245 | 0.3596 | Yes |
| 59 | P4HA1 |  |  | 792 | 0.242 | 0.3626 | Yes |
| 60 | TSPAN3 |  |  | 822 | 0.237 | 0.3634 | Yes |
| 61 | PLEC |  |  | 824 | 0.237 | 0.3674 | Yes |
| 62 | NT5E |  |  | 834 | 0.235 | 0.3705 | Yes |
| 63 | GPRC5B |  |  | 838 | 0.234 | 0.3743 | Yes |
| 64 | SPATA6 |  |  | 852 | 0.232 | 0.3769 | Yes |
| 65 | COMT |  |  | 938 | 0.220 | 0.3710 | Yes |
| 66 | HES4 |  |  | 977 | 0.213 | 0.3704 | Yes |
| 67 | MAGT1 |  |  | 1006 | 0.210 | 0.3709 | Yes |
| 68 | PLTP |  |  | 1015 | 0.209 | 0.3736 | Yes |
| 69 | DKK3 |  |  | 1050 | 0.204 | 0.3733 | Yes |
| 70 | C1orf61 |  |  | 1057 | 0.204 | 0.3762 | Yes |
| 71 | CYP51A1 |  |  | 1061 | 0.203 | 0.3794 | Yes |
| 72 | LEPROT |  |  | 1072 | 0.202 | 0.3818 | Yes |
| 73 | SEMA6A |  |  | 1075 | 0.202 | 0.3851 | Yes |
| 74 | TIMP3 |  |  | 1191 | 0.186 | 0.3752 | No |
| 75 | TFPI |  |  | 1278 | 0.175 | 0.3685 | No |
| 76 | SEMA6D |  |  | 1286 | 0.173 | 0.3707 | No |
| 77 | ELOVL5 |  |  | 1320 | 0.169 | 0.3699 | No |
| 78 | IFITM3 |  |  | 1332 | 0.168 | 0.3716 | No |
| 79 | CTSL |  |  | 1384 | 0.163 | 0.3686 | No |
| 80 | GJA1 |  |  | 1399 | 0.162 | 0.3699 | No |
| 81 | SOAT1 |  |  | 1400 | 0.162 | 0.3727 | No |
| 82 | BMP7 |  |  | 1419 | 0.160 | 0.3734 | No |
| 83 | IFI27L1 |  |  | 1481 | 0.154 | 0.3692 | No |
| 84 | LIFR |  |  | 1520 | 0.150 | 0.3675 | No |
| 85 | CST3 |  |  | 1602 | 0.141 | 0.3607 | No |
| 86 | SPARCL1 |  |  | 1663 | 0.136 | 0.3562 | No |
| 87 | PAQR8 |  |  | 1666 | 0.136 | 0.3584 | No |
| 88 | CSPG5 |  |  | 1670 | 0.136 | 0.3604 | No |
| 89 | MEIS1 |  |  | 1728 | 0.131 | 0.3562 | No |
| 90 | LRRC17 |  |  | 1859 | 0.122 | 0.3435 | No |
| 91 | HMGCS1 |  |  | 1899 | 0.118 | 0.3411 | No |
| 92 | CCDC80 |  |  | 1921 | 0.117 | 0.3408 | No |
| 93 | TNFRSF19 |  |  | 1943 | 0.116 | 0.3404 | No |
| 94 | NLGN3 |  |  | 1947 | 0.115 | 0.3421 | No |
| 95 | NOTCH3 |  |  | 1978 | 0.113 | 0.3406 | No |
| 96 | WSCD1 |  |  | 1980 | 0.113 | 0.3425 | No |
| 97 | SLC1A4 |  |  | 2049 | 0.108 | 0.3366 | No |
| 98 | TSPAN6 |  |  | 2087 | 0.106 | 0.3343 | No |
| 99 | CD82 |  |  | 2145 | 0.102 | 0.3295 | No |
| 100 | GDPD2 |  |  | 2263 | 0.094 | 0.3178 | No |
| 101 | HES1 |  |  | 2302 | 0.092 | 0.3151 | No |
| 102 | GRB14 |  |  | 2398 | 0.086 | 0.3057 | No |
| 103 | LRP4 |  |  | 2410 | 0.084 | 0.3060 | No |
| 104 | LIPA |  |  | 2451 | 0.082 | 0.3028 | No |
| 105 | PHGDH |  |  | 2485 | 0.081 | 0.3005 | No |
| 106 | P3H4 |  |  | 2488 | 0.080 | 0.3017 | No |
| 107 | PBXIP1 |  |  | 2536 | 0.077 | 0.2976 | No |
| 108 | EFEMP2 |  |  | 2557 | 0.076 | 0.2967 | No |
| 109 | ADORA2B |  |  | 2563 | 0.076 | 0.2975 | No |
| 110 | BBS2 |  |  | 2698 | 0.068 | 0.2833 | No |
| 111 | ITPR2 |  |  | 2803 | 0.063 | 0.2726 | No |
| 112 | GPR137B |  |  | 2815 | 0.062 | 0.2724 | No |
| 113 | PHYHIPL |  |  | 2821 | 0.062 | 0.2729 | No |
| 114 | ETV1 |  |  | 2867 | 0.059 | 0.2688 | No |
| 115 | CA12 |  |  | 2885 | 0.058 | 0.2679 | No |
| 116 | ETV5 |  |  | 2957 | 0.055 | 0.2607 | No |
| 117 | ABAT |  |  | 3020 | 0.051 | 0.2545 | No |
| 118 | ARHGEF26 |  |  | 3032 | 0.050 | 0.2542 | No |
| 119 | PPP1R16A |  |  | 3059 | 0.049 | 0.2521 | No |
| 120 | SLC38A3 |  |  | 3125 | 0.046 | 0.2454 | No |
| 121 | C2orf72 |  |  | 3156 | 0.044 | 0.2428 | No |
| 122 | DTNA |  |  | 3219 | 0.040 | 0.2364 | No |
| 123 | RAMP1 |  |  | 3221 | 0.040 | 0.2370 | No |
| 124 | TOR1AIP1 |  |  | 3313 | 0.036 | 0.2272 | No |
| 125 | SLC25A18 |  |  | 3352 | 0.034 | 0.2235 | No |
| 126 | TCF7L2 |  |  | 3356 | 0.033 | 0.2237 | No |
| 127 | C3orf70 |  |  | 3474 | 0.028 | 0.2109 | No |
| 128 | RGMA |  |  | 3522 | 0.026 | 0.2060 | No |
| 129 | LRRN3 |  |  | 3720 | 0.019 | 0.1838 | No |
| 130 | GULP1 |  |  | 3837 | 0.014 | 0.1708 | No |
| 131 | TECPR2 |  |  | 4057 | 0.005 | 0.1459 | No |
| 132 | BDH2 |  |  | 4092 | 0.004 | 0.1421 | No |
| 133 | EEPD1 |  |  | 4100 | 0.003 | 0.1413 | No |
| 134 | SIRPA |  |  | 4174 | -0.000 | 0.1330 | No |
| 135 | ROBO3 |  |  | 4187 | -0.001 | 0.1316 | No |
| 136 | GNPTG |  |  | 4264 | -0.004 | 0.1230 | No |
| 137 | SOX2 |  |  | 4271 | -0.005 | 0.1224 | No |
| 138 | SOX8 |  |  | 4306 | -0.006 | 0.1187 | No |
| 139 | RASSF4 |  |  | 4325 | -0.007 | 0.1167 | No |
| 140 | DARS |  |  | 4329 | -0.007 | 0.1165 | No |
| 141 | CAMK2G |  |  | 4597 | -0.018 | 0.0863 | No |
| 142 | SERPINB6 |  |  | 4759 | -0.024 | 0.0684 | No |
| 143 | FAM181B |  |  | 4789 | -0.025 | 0.0655 | No |
| 144 | ID4 |  |  | 4830 | -0.027 | 0.0614 | No |
| 145 | SPRY1 |  |  | 4844 | -0.027 | 0.0604 | No |
| 146 | ABCD3 |  |  | 5045 | -0.034 | 0.0382 | No |
| 147 | MYO10 |  |  | 5109 | -0.037 | 0.0316 | No |
| 148 | APBB2 |  |  | 5184 | -0.039 | 0.0239 | No |
| 149 | KLHDC8A |  |  | 5283 | -0.043 | 0.0134 | No |
| 150 | ATP1B2 |  |  | 5376 | -0.048 | 0.0038 | No |
| 151 | PHACTR2 |  |  | 5613 | -0.058 | -0.0221 | No |
| 152 | FERMT2 |  |  | 5664 | -0.061 | -0.0268 | No |
| 153 | ALDH6A1 |  |  | 5682 | -0.061 | -0.0277 | No |
| 154 | QKI |  |  | 5715 | -0.062 | -0.0302 | No |
| 155 | EMC2 |  |  | 5831 | -0.067 | -0.0422 | No |
| 156 | SASH1 |  |  | 5850 | -0.067 | -0.0431 | No |
| 157 | PDLIM3 |  |  | 5938 | -0.071 | -0.0517 | No |
| 158 | SHISA4 |  |  | 5957 | -0.072 | -0.0525 | No |
| 159 | ADD3 |  |  | 5984 | -0.073 | -0.0542 | No |
| 160 | GATM |  |  | 6035 | -0.076 | -0.0586 | No |
| 161 | CRYL1 |  |  | 6120 | -0.081 | -0.0668 | No |
| 162 | PDLIM5 |  |  | 6127 | -0.081 | -0.0660 | No |
| 163 | SLC9A3R1 |  |  | 6276 | -0.089 | -0.0814 | No |
| 164 | YIF1A |  |  | 6359 | -0.093 | -0.0891 | No |
| 165 | SNX5 |  |  | 6418 | -0.097 | -0.0940 | No |
| 166 | CDC42EP4 |  |  | 6451 | -0.099 | -0.0960 | No |
| 167 | TRIM47 |  |  | 6510 | -0.102 | -0.1008 | No |
| 168 | GNG7 |  |  | 6584 | -0.107 | -0.1073 | No |
| 169 | TSC22D4 |  |  | 6599 | -0.108 | -0.1070 | No |
| 170 | TP53I3 |  |  | 6607 | -0.108 | -0.1059 | No |
| 171 | OAT |  |  | 6648 | -0.111 | -0.1085 | No |
| 172 | CDC42EP1 |  |  | 6781 | -0.119 | -0.1215 | No |
| 173 | PREX1 |  |  | 6878 | -0.128 | -0.1302 | No |
| 174 | ZFYVE21 |  |  | 7098 | -0.145 | -0.1526 | No |
| 175 | MGLL |  |  | 7100 | -0.145 | -0.1502 | No |
| 176 | GFAP |  |  | 7113 | -0.146 | -0.1490 | No |
| 177 | EFHD2 |  |  | 7122 | -0.147 | -0.1474 | No |
| 178 | HADHB |  |  | 7132 | -0.148 | -0.1458 | No |
| 179 | DDAH1 |  |  | 7257 | -0.162 | -0.1571 | No |
| 180 | ZFP36L2 |  |  | 7317 | -0.169 | -0.1609 | No |
| 181 | ID2 |  |  | 7333 | -0.171 | -0.1596 | No |
| 182 | IDI1 |  |  | 7337 | -0.172 | -0.1569 | No |
| 183 | HEY1 |  |  | 7370 | -0.177 | -0.1575 | No |
| 184 | RGS20 |  |  | 7387 | -0.180 | -0.1562 | No |
| 185 | SPRY2 |  |  | 7461 | -0.187 | -0.1612 | No |
| 186 | ZFP36L1 |  |  | 7466 | -0.188 | -0.1584 | No |
| 187 | PLEKHB1 |  |  | 7506 | -0.191 | -0.1595 | No |
| 188 | UBL3 |  |  | 7525 | -0.194 | -0.1581 | No |
| 189 | GLUD1 |  |  | 7545 | -0.197 | -0.1569 | No |
| 190 | GLUL |  |  | 7594 | -0.203 | -0.1588 | No |
| 191 | OLFM2 |  |  | 7624 | -0.207 | -0.1585 | No |
| 192 | PSAT1 |  |  | 7709 | -0.220 | -0.1642 | No |
| 193 | DCXR |  |  | 7713 | -0.220 | -0.1607 | No |
| 194 | TRIB2 |  |  | 7741 | -0.224 | -0.1599 | No |
| 195 | AIF1L |  |  | 7762 | -0.227 | -0.1582 | No |
| 196 | ID1 |  |  | 7821 | -0.238 | -0.1606 | No |
| 197 | JUN |  |  | 7930 | -0.262 | -0.1684 | No |
| 198 | RAB34 |  |  | 8218 | -0.330 | -0.1953 | No |
| 199 | HOPX |  |  | 8257 | -0.341 | -0.1937 | No |
| 200 | MT3 |  |  | 8279 | -0.348 | -0.1900 | No |
| 201 | LITAF |  |  | 8324 | -0.361 | -0.1887 | No |
| 202 | SOX9 |  |  | 8327 | -0.361 | -0.1826 | No |
| 203 | GNG12 |  |  | 8328 | -0.362 | -0.1763 | No |
| 204 | PLIN3 |  |  | 8403 | -0.388 | -0.1779 | No |
| 205 | ENHO |  |  | 8432 | -0.400 | -0.1741 | No |
| 206 | CBR1 |  |  | 8480 | -0.424 | -0.1721 | No |
| 207 | PEA15 |  |  | 8509 | -0.439 | -0.1676 | No |
| 208 | LGALS3 |  |  | 8550 | -0.460 | -0.1641 | No |
| 209 | HSPB1 |  |  | 8586 | -0.478 | -0.1597 | No |
| 210 | ANXA5 |  |  | 8626 | -0.510 | -0.1552 | No |
| 211 | VIM |  |  | 8663 | -0.535 | -0.1500 | No |
| 212 | C1orf122 |  |  | 8686 | -0.548 | -0.1429 | No |
| 213 | S100A16 |  |  | 8739 | -0.596 | -0.1384 | No |
| 214 | MT2A |  |  | 8740 | -0.598 | -0.1279 | No |
| 215 | CNN3 |  |  | 8752 | -0.613 | -0.1184 | No |
| 216 | FABP7 |  |  | 8811 | -0.661 | -0.1135 | No |
| 217 | GNG11 |  |  | 8815 | -0.665 | -0.1022 | No |
| 218 | S100A13 |  |  | 8820 | -0.670 | -0.0909 | No |
| 219 | RHOC |  |  | 8823 | -0.676 | -0.0793 | No |
| 220 | FABP5 |  |  | 8829 | -0.684 | -0.0679 | No |
| 221 | RAB31 |  |  | 8831 | -0.687 | -0.0559 | No |
| 222 | GNG5 |  |  | 8866 | -0.728 | -0.0471 | No |
| 223 | SNX3 |  |  | 8881 | -0.748 | -0.0356 | No |
| 224 | DBI |  |  | 8895 | -0.764 | -0.0237 | No |
| 225 | S100B |  |  | 8908 | -0.784 | -0.0113 | No |
| 226 | LGALS1 |  |  | 8982 | -1.135 | 0.0002 | No |
Table: GSEA details [plain text format]

  

Fig 2: RICHARDS\_ZHONG\_NATURE\_2018\_ASTROCYTES\_UPREG: Random ES distribution      
 Gene set null distribution of ES for **RICHARDS\_ZHONG\_NATURE\_2018\_ASTROCYTES\_UPREG**
